# EXAMINING THE FEASIBILITY AND BENEFITS OF SELF-QUANTIFICATION FOR FAMILY CAREGIVERS: A MIXED-METHOD APPROACH

**DOI:** 10.1093/geroni/igae098.3129

**Published:** 2024-12-31

**Authors:** Tomoko Wakui, Suguru Okubo, Satoko Fujihara, Yoko Moriyama, Takeshi Nakagawa, Shuichi Obuchi, Shuichi Awata, Ichiro Kai

**Affiliations:** Tokyo Metropolitan Institute for Geriatrics and Gerontology, Tokyo, Japan; BMS Yokohama Inc., Yokohama, Kanagawa, Japan; Tokyo Metropolitan Institute for Geriatrics and Gerontology, Tokyo, Japan; National Institute of Public Health, Wako, Saitama, Japan; National Center for Geriatrics and Gerontology, Obu, Aichi, Japan; Tokyo Metropolitan Institute for Geriatrics and Gerontology, Tokyo, Japan; Tokyo Metropolitan Institute for Geriatrics and Gerontology, Tokyo, Japan; University of Tokyo, Tokyo, Japan

## Abstract

Supporting family caregivers poses significant challenges because of their inability to seek assistance amid limited time and burden. This study aimed to explore the feasibility of implementing a self-quantification program for family caregivers of community-dwelling older adults. We developed the Caregiving Visualization Program (Care-VIP) allowing family caregivers to document their daily caregiving and life activities and emotional well-being. Initially, an online survey was conducted with 3,256 family caregivers, who were proportionately distributed based on the percentage of the nation’s older population requiring care, to investigate their caregiving situations. Subsequently, 201 participants, who requested the program participated, engaged in the self-quantification program for 60 days and provided feedback in a follow-up survey. Feasibility was evaluated based on participant characteristics, completion rates, and the qualitative benefits of self-quantification. Logistic regression analysis revealed that participants were more likely to be fully employed (OR:1.79; p<.01) and experience higher levels of burden (OR:1.01; p<.01) and daily caregiving demands (OR:1.50; p<.01). The completion rate after 60 days was 87.9%, with a 90% completion rate at 98.5%. The content analysis of open-ended responses identified several benefits, including “purposeful and attentive care”, “identifying improvements”, “establishing daily routines”, and “enhancing emotional and self-awareness” derived from “systematic care analysis” facilitated by “reflection”. Furthermore, self-quantification led to “instrumental record-keeping”, encouraging “articulated insight,” fostering “information sharing”. This study discusses the significance and limitations of reflective practices and documentation activities with Care-VIP in supporting more frequent and heavily burdened family caregivers.

